# Lung Cancer in a U.S. Population with Low to Moderate Arsenic Exposure

**DOI:** 10.1289/ehp.0900566

**Published:** 2009-07-02

**Authors:** Julia E. Heck, Angeline S. Andrew, Tracy Onega, James R. Rigas, Brian P. Jackson, Margaret R. Karagas, Eric J. Duell

**Affiliations:** 1 International Agency for Research on Cancer, Lyon, France; 2 School of Public Health and Jonsson Comprehensive Cancer Center, University of California, Los Angeles, California, USA; 3 Norris Cotton Cancer Center and; 4 Section of Biostatistics and Epidemiology, Department of Community and Family Medicine, Dartmouth Medical School, Lebanon, New Hampshire, USA; 5 Comprehensive Thoracic Oncology Program, Dartmouth-Hitchcock Medical Center, Lebanon, New Hampshire, USA; 6 Department of Earth Sciences, Dartmouth College, Hanover, New Hampshire, USA

**Keywords:** arsenic, bronchitis, chronic obstructive pulmonary disease, lung cancer, lung diseases, New Hampshire, pulmonary fibrosis, small-cell carcinoma, smoking, Vermont

## Abstract

**Background:**

Little is known about the carcinogenic potential of arsenic in areas with low to moderate concentrations of arsenic (< 100 μg/L) in drinking water.

**Objectives:**

We examined associations between arsenic and lung cancer.

**Methods:**

A population-based case–control study of primary incident lung cancer was conducted in 10 counties in two U.S. states, New Hampshire and Vermont. The study included 223 lung cancer cases and 238 controls, each of whom provided toenail clippings for arsenic exposure measurement by inductively coupled–plasma mass spectrometry. We estimated odds ratios (ORs) of the association between arsenic exposure and lung cancer using unconditional logistic regression with adjustment for potential confounders (age, sex, race/ethnicity, smoking pack-years, education, body mass index, fish servings per week, and toenail selenium level).

**Results:**

Arsenic exposure was associated with small-cell and squamous-cell carcinoma of the lung [OR = 2.75; 95% confidence interval (CI), 1.00–7.57] for toenail arsenic concentration ≥ 0.114 μg/g, versus < 0.05 μg/g. A history of lung disease (bronchitis, chronic obstructive pulmonary disease, or fibrosis) was positively associated with lung cancer (OR = 2.86; 95% CI, 1.39–5.91). We also observed an elevated risk of lung cancer among participants with a history of lung disease and toenail arsenic ≥ 0.05 μg/g (OR = 4.78; 95% CI, 1.87–12.2) than among individuals with low toenail arsenic and no history of lung disease.

**Conclusion:**

Although this study supports the possibility of an increased risk of specific lung cancer histologic types at lower levels of arsenic exposure, we recommend large-scale population-based studies.

Arsenic in drinking water is a major environmental carcinogen. Worldwide, millions of people suffer debilitating health effects from inorganic arsenic exposure, including cancer and vascular, pulmonary, hematologic, neurologic, and developmental disorders [Heck et al. 2008a; International Agency for Research on Cancer (IARC) 2004]. In the United States, an estimated 13 million people are exposed to arsenic concentrations that exceed the U.S. Environmental Protection Agency’s (EPA) maximum contaminant level of 10 ppb (U.S. EPA 2001).

An increase in the incidence of skin, bladder, and lung cancers at high arsenic concentrations is well established (IARC 2004). However, the cancer risk from exposure to lower levels (< 100 μg/L) of arsenic is largely unknown. The results from other studies have been inconsistent (Ahsan et al. 2000; Chen et al. 2004; Ferreccio et al. 1998; Karagas et al. 2001, 2002; Lamm et al. 2004; Lewis et al. 1999), perhaps due, in part, to exposure variation in settings where people have access to noncontaminated water sources. Inconsistencies in results may also be related to a lack of information on individual cofactors, such as smoking or relevant health conditions, or to regional differences in factors associated with arsenic susceptibility, such as nutrition (Heck et al. 2007, 2009).

Lung cancer is the leading cause of cancer- related mortality in the United States and worldwide. IARC (2004) has classified arsenic as a group 1 carcinogen for lung cancer (IARC 2004). This assessment was based on studies in which arsenic exposure was inferred by using area of residence or the arsenic concentration the in well water rather than using an individual biomarker of exposure (Chen et al. 1985, 1986, 1988a, 1988b; Chen and Wang 1990; Chiou et al. 1995; Ferreccio et al. 2000; Hinwood et al. 1999; Hopenhayn-Rich et al. 1998; Lewis et al. 1999; Nakadaira et al. 2002; Rivara et al. 1997; Smith et al. 1998; Tsai et al. 1999; Tsuda et al. 1995; Wu et al. 1989). The studies not included in the IARC evaluation and those that have been published since also have been based on local or regional well-water concentrations (Baastrup et al. 2008; Chen et al. 2004; Ferreccio et al. 1998; Guo 2004; Han et al. 2008; Marshall et al. 2007; Mostafa et al. 2008; Smith et al. 2006).

The use of a biomarker of arsenic exposure may help to improve the assessment of low-dose health effects, including cancer incidence (Karagas et al. 2002). Trivalent inorganic arsenic binds to the sulfhydryl groups in nail keratin cells and thus makes toenail arsenic a reasonable measure of arsenic exposure. Depending on the toe and the speed of nail growth, toenail measurements represent exposures that occurred 3–12 months before sample collection. This finding has been found to be relatively stable over time (Garland et al. 1993). In this study, we used toenail arsenic concentration as a biomarker of exposure to examine the risk of lung cancer among persons in the U.S. population who had been exposed to low levels of arsenic in drinking water.

## Materials and Methods

The New England Lung Cancer Study (NELCS), a population-based case–control study of lung cancer, was conducted in seven New Hampshire counties (Belknap, Carroll, Cheshire, Coos, Grafton, Merrimack, and Sullivan) and in three Vermont counties (Orange, Windham, and Windsor). We used the New Hampshire State Cancer Registry, the Dartmouth-Hitchcock Tumor Registry of the Norris Cotton Cancer Center, and the Dartmouth-Hitchcock Medical Center in Lebanon, New Hampshire, to identify persons from 2005 to 2007 who had received a clinical diagnosis of lung cancer. We obtained the names of cases within 1 to 6 months of their initial diagnosis. Cases who had histologically confirmed primary incident lung cancer (World Health Organization 2000), were between 30 and 74 years of age, resided in one of the 10 study counties, were alive at first contact, had a working telephone number, and were able to communicate in English. Of the 454 eligible cases, 24 (5%) could not be reached because of an inaccurate address or phone number, 52 (11%) were too ill to be interviewed, and 101 (22%) refused to participate, which yielded 277 subjects and a 61% participation rate.

Control participants were identified using a commercial database from Experian for the contiguous 10-county study area for the period 2005–2006. For each potential control, the database contained the full name, date of birth (or estimated age), address, sex, and telephone number. Using a marginal count comparison with U.S. Census Bureau data for the same time period, we identified 73.4% of women and 72.4% of men in the NELCS study area in the commercial database. The eligibility of the controls was based on the same criteria as that used for cases except for the presence of lung cancer. Controls were randomly selected from the commercial database and frequency matched to lung cancer cases within 5-year age group and by sex. Of the 547 eligible controls, 123 (22%) could not be reached because of inaccurate address or phone number, 11 (2%) were too ill to be interviewed, and 162 (30%) refused participation. A total of 251 controls completed the interview for a 46% participation rate. Because self-reported home ownership was somewhat higher among controls than among cases, we ran all models with and without a variable for home ownership as well as educational attainment and household income to account for potential differences in these socioeconomic variables.

### Study protocol

A letter describing the study was sent to potential cases and controls informing them of the study and mentioning that a study interviewer would be contacting them by telephone within 2 weeks. A prepaid postcard was included that requested an updated phone number if the number provided was no longer valid. At the initial telephone contact, the individual’s name and age were confirmed, verbal permission obtained, and an interview date requested. At the in-person interview, written informed consent was obtained from all participants before participation. The study complied with all applicable requirements of the Dartmouth College’s Committee for the Protection of Human Subjects, the Norris Cotton Cancer Center, and the State of New Hampshire for obtaining case names from the New Hampshire State Cancer Registry. Most case and control interviews occurred in the participant’s home; some cases were interviewed in the Norris Cotton Cancer Center or other location at the participant’s request. All in-person interviews were conducted by trained interviewers using a structured questionnaire on demographic, lifestyle and medical history; a 121-item, validated food frequency questionnaire (Salvini et al. 1989); and a lifetime residential history calendar. The following biological specimens were requested at the interview: a 20-mL blood sample (32% of blood samples from cases were obtained before initiation of therapy), toenail clippings, an oral buccal brushing, and a Scope (Procter & Gamble Co., Cincinnati, OH, USA) mouthwash sample, which was used as a source of oral cells for genomic DNA isolation from those unable or unwilling to provide a blood sample. In this study, we used only toenail samples and questionnaire data for the arsenic analysis.

Of the 528 participants, we excluded from the analysis 62 who did not provide a toenail sample and 2 (< 1%) whose toenail selenium could not be analyzed. An additional 3 subjects (< 1%) were excluded because of missing information on smoking or weight. The final sample size was 461 participants.

### Toenail arsenic analyses

Toenails were thoroughly washed (and sonicated) with acetone and Triton-X 100 and rinsed five times in deionized water to remove exogenous contamination. The nail sample was acid digested in HNO_3_ (Optima; Fisher Scientific, St. Louis, MO, USA) using microwave techniques (Microwave Accelerated Reaction System 5; CEM Corp., Mathews, NC, USA).

Diluted aliquots of the digested toe-nail samples were analyzed by collision cell inductively coupled plasma–mass spectrometry (7500c, Agilent Technologies, Inc., Santa Clara, CA, USA). Quality control included assessing the percent recovery of the GBW 07601 hair standard reference material (National Analysis Center, Beijing, China) with a certified arsenic value of 0.28 ± 0.04 mg/kg. Our repeated analysis of this reference material (*n* = 40 > 2 years) gave a value of 0.235 ± 0.038 (2σ). We also ran repeated analyses of a second source matrix-spiked sample (as an initial calibration check and a continuing calibration check) at a frequency of every 10 samples and control criteria of 80–120% recovery. Analysis blanks were run at a frequency of every 20 samples. Digestion blanks were routinely analyzed and were generally low (< 10 ng/L in the 4× diluted samples). The mean plus 3 times the standard deviation (SD) of the digestion blanks was used to determine method detection. The detection limits are a function of both the method detection limit and the mass of the nail available for the digestion. The average sample specific detection limit for arsenic was 0.003 μg/g. For four persons, the measured arsenic fell below this limit, and these individuals were assigned an arsenic value of 0.0015.

### Statistical analyses

We analyzed the demograhic and health characteristics of the cases and controls and report the odds ratios (ORs) of the diseases associated with these characteristics. Geometric means of toenail arsenic concentrations were determined and compared using analysis of variance. Arsenic exposure has been linked to bronchitis, chronic obstructive pulmonary disease (COPD), fibrosis, diabetes, and cardiovascular disease (IARC 2004; Mazumder et al. 2005; Smith et al. 2006; States et al. 2009; von Ehrenstein et al. 2005). To describe any potential risk for these conditions, we provided geometric means for toenail arsenic concentrations among persons with and without these diseases. To describe the possible arsenic sources in this population, we also determined arsenic concentrations from drinking water sources and from other potential exposure routes such as previous employment in farmwork or woodworking.

We used unconditional logistic regression analyses to examine the relative risk of lung cancer with toenail arsenic levels. In the main analyses, we categorized toenail arsenic into four levels: < 0.05 μg/g, 0.05 to < 0.0768 μg/g, 0.768 to < 0.1137 μg/g, and ≥ 0.1137 μg/g. The rationale for this stratification was based on results from a study of well water and toenail arsenic from participants in the same region where Karagas et al. (2000) observed that individuals with toenail arsenic < 0.05 μg/g were almost uniformly exposed to drinking water arsenic concentrations of < 1 μg/L. Above that point, we categorized the participants into three levels based on the arsenic concentrations among the controls. Because arsenic has been reported to be associated with squamous-cell and small-cell carcinoma, we also analyzed lung cancer risk by histology (Guo et al. 2004; Mostafa et al. 2008).

Variables considered for inclusion in the logistic regression models were those previously associated with arsenic, with lung cancer incidence, and with interindividual variation in nail growth (Ahsan et al. 2006; Slotnick and Nriagu 2006). The analyses also included age, sex, race/ethnicity, tobacco smoking (pack-years), educational attainment (< high school, high school, > high school), body mass index (BMI: < 18.5, 18.5 to < 25, ≥ 25), prior self-reported history of arsenic-related lung disease (bronchitis, COPD, or fibrosis), servings of fish per week, and selenium intake (quartiles of toenail concentration; milligrams per kilogram). We included selenium in the model because it has been linked to cancer prevention in the presence of arsenic (Chen et al. 2007). Fish have trace levels of arsenic, mostly in organic form, but may also have trace levels of inorganic arsenic, which in humans, has been linked to tobacco use and to toenail arsenic concentration (Heck et al. 2008b; Slotnick and Nriagu 2006). Eleven subjects did not complete the food frequency questionnaire and were assigned a weekly fish intake equivalent to the mean in the overall sample (mean, 1.75 servings).

Because of potential synergistic effects between arsenic and smoking and between arsenic and lung disease, we present the results of models that examined these possible interaction effects (Chen et al. 2004; Parvez et al. 2008). We also examined whether a history of asthma was associated with arsenic and lung cancer; we found no association and left this variable out of final models. We also observed no evidence of an interaction effect between arsenic and selenium on lung cancer risk (*p*= 0.4).

## Results

Study subjects were similar in age and sex (Table 1). The mean age (± SD) was 61.9 ± 9.1 years for cases and 61.0 ± 10.2 years for controls. Controls smoked less than cases, and more controls than cases had received education beyond high school. Toenail arsenic concentrations for the entire study population ranged from 0.007 to 1.57 μg/g. The distribution of toenail arsenic concentrations was skewed: 29.1% had arsenic at < 0.05 μg/g, 42.3% between 0.05 and 0.1 μg/g, 18.4% between 0.1 and 0.15 μg/g, 5.2% between 0.15 and 0.2 μg/g, 4.6% between 0.2 and 0.7 μg/g, and < 1% (two subjects) had arsenic > 1 μg/g. Toenail arsenic concentrations differed by age, with the highest concentrations among persons < 70 years of age (Table 1). Toenail arsenic concentration was positively associated with fish servings per week and in controls, it was positively associated with toenail selenium concentration. The average length of time at the current residence did not differ between cases (mean, 17.2 years) and controls (mean, 16.9 years).

In Table 2, we describe prior lung disease, cardiovascular disease, adult-onset diabetes, and potential sources of arsenic exposure in the study population in relation to mean arsenic levels. Among controls, those who received their water from a nonfiltered well or spring had higher mean toenail arsenic concentrations than did those who drank filtered water. Cases who owned their own homes had higher mean arsenic than did cases who were not homeowners.

In the multiple regression analyses, higher toenail arsenic levels were associated with small-cell carcinoma and squamous-cell carcinoma lung cancer (Table 3). We also observed an increased risk of lung cancer among those who reported a prior history of lung disease (OR = 3.2). With stratification by histology, we noted that an increased risk of lung cancer was driven primarily by small-cell carcinoma cases, although the number of cases in each histologic group was small (data not shown).

When we examined all lung cancers combined, we found no evidence of an interaction effect between arsenic exposure and cigarette smoking in relation to lung cancer risk (Table 4). When we stratified by self- reported history of lung disease, we observed an increased risk of lung cancer among patients with lung disease and toenail arsenic concentrations > 0.05 μg/g. We obtained similar results when we excluded the two patients with toenail arsenic > 1 μg/g (data not shown).

Because home ownership was more common among the cases in the Experian database (80%) than among the cases in the study area (by county, 68–78%) (U.S. Census Bureau 2008), we examined whether effect estimates were similar when we limited the analyses to homeowners (*n* = 388, 85%), which yielded similar results to those seen in the overall findings (data not shown).

## Discussion

For persons in this study population who were exposed to low to moderate levels of arsenic from drinking water, we observed an increased risk of small-cell carcinoma and squamous-cell carcinoma lung cancer among participants with higher arsenic concentrations in toe-nails. Because of the small sample size of this study, we recommend that our findings be interpreted with caution. Not all studies have observed a varying presentation of histologic types in the presence of arsenic (Chen et al. 2004). However, a higher risk of squamous-cell carcinoma was observed in Bangladesh, a region with high concentrations of arsenic in the drinking water (up to 366 μg/L) (Mostafa et al. 2008). Several case reports, occupational studies, and a published study on drinking water from Taiwan have all linked arsenic exposure to small-cell carcinoma of the lung (Guo et al. 2004; Heddle and Bryant 1983; Kusiak et al. 1993; Lee and Bebb 2005; Pershagen et al. 1987).

Because we did not collect samples of each participant’s drinking water, we were unable to report the risk associated with specific arsenic concentrations in well water. A geologic survey of the region found that 28% of private wells in New Hampshire and 6.7% of wells in Vermont have arsenic concentrations > 5 μg/L (Ayotte et al. 2006). Toenail arsenic has been correlated with well-water arsenic concentrations in New Hampshire and in areas with higher levels of arsenic (Karagas et al. 2000; Kile et al. 2007). At lower doses, toenail arsenic concentrations are less likely to be correlated with concentrations of arsenic in drinking water due, most likely, to contributions from other exposure sources. A separate investigation of subjects from the same region found that subjects with toenail arsenic between 0.05 and 0.5 μg/g had water arsenic concentrations between 1 and 100 μg/L (Karagas et al. 2000). Besides drinking water exposure, subjects also may have been exposed to trace amounts of arsenic from dietary sources, tobacco, or airborne particle inhalation. We do not anticipate strong effects from these other sources, because we accounted for some other exposure sources, such as fish and smoking, in our multivariate analysis.

We also observed an increased risk of lung cancer among participants who reported a prior history of nonmalignant lung disease (bronchitis, COPD, or fibrosis). A number of previous studies have linked arsenic exposure to chronic lung diseases, including shortness of breath, chest sounds, chronic bronchitis, bronchiectasis, COPD, and interstitial fibrosis (Guha Mazumder 2007; Mazumder et al. 2005; Milton and Rahman 2002; Milton et al. 2003; Smith et al. 2006; States et al. 2009). The reported concentrations of water arsenic concentrations in these studies were as high as > 300 μg/L (Mazumder et al. 2005), > 400 μg/L (Guha Mazumder 2007), or 1,000 μg/L (Milton and Rahman 2002). In developing country settings with a wide range of arsenic concentrations in drinking water, dose–response effects have been observed between arsenic levels and chronic cough among both smokers and nonsmokers (Guha Mazumder 2007; Smith et al. 2006). Although drinking water arsenic increases both lung disease and lung cancer rates within the same population (Smith et al. 2006), to our knowledge, no previous studies have examined a potential synergistic effect, perhaps due in part to the ecologic design of most studies. Differential effects have been observed in inhalation studies. For example, Chen and Chen (2002) found that Chinese tin miners with silicosis who were exposed to arsenic at three mines had an increased risk of lung cancer, but not at a fourth mine where arsenic concentrations were lower. In a study of inhaled arsenic, Taeger et al. (2009) found that silicosis appeared to be related to the cell type of lung cancer among the uranium workers who were exposed to arsenic. However, it is difficult to draw conclusions from these studies because the mechanism of arsenic-related lung diseases and lung cancer may differ when arsenic is inhaled rather than ingested.

We did not observe an independent association between arsenic and lung disease in our cases, suggesting that the possible synergistic effect should be interpreted with caution. In other studies, the presence of respiratory symptoms has been reported to be 10–25 times more common among persons with arsenical dermatosis, even compared with healthy persons living in the same region who are likely exposed to similar arsenic concentrations in water (Borgono et al. 1977; De et al. 2004; Guha Mazumder 2007; Mazumder et al. 2005; Milton and Rahman 2002; Milton et al. 2003). We cannot rule out the possibility that increased reporting of prior lung disease among cases (recall bias) may in part explain these results and ours. Nevertheless, in a small study, decreases in forced expiratory volume and forced vital capacity were more pronounced among persons with arsenic- related skin cancers than among cancer-free controls who also were exposed to arsenic in the drinking water (von Ehrenstein et al. 2005). However, the greater arsenic concentrations in those studies make it difficult to directly compare with our results. We recommend further studies in other populations exposed to low or moderate levels of arsenic.

Further research is needed to better understand the biologic mechanism by which arsenic affects lung function and lung cancer. Circulating arsenic is known to be deposited in the lung, particularly in epithelial tissue. Arsenic has been associated with both obstructive and restrictive changes in pulmonary function (Guha Mazumder 2007). Olsen et al. (2008) found evidence for increased lung inflammation and inhibition of wound repair even at low levels of arsenic exposure. As a carcinogen, mechanisms posited for arsenic include genetic and epigenetic changes, inhibition of DNA repair, oxidative stress, apoptosis, and modulation of signal transduction pathways (Andrew et al. 2006; Huang et al. 2004).

In contrast to other studies (Chen et al. 2004; Mostafa et al. 2008), we observed no interaction between smoking and arsenic in lung cancer risk. This difference may be explained by the considerably lower arsenic concentrations seen in the NELCS area. In the study by Mostafa et al. (2008), risk estimates for lung cancer did not differ between smokers and nonsmokers when arsenic concentrations were > 100 μg/L.

A strength of this investigation was the population-based study design and data collection by in-person interview, with detailed information sought about prior medical history. To our knowledge this study was one of the first on arsenic and lung cancer that measured arsenic exposure using a biomarker of exposure. In developed nations, individuals exposed to arsenic in drinking water are likely to have access to other water sources. Thus, ecologic analyses of arsenic concentrations in drinking water and cancer are more likely subject to the ecologic fallacy than are studies in developing countries.

A limitation of this study was the response rate difference between cases and controls, which would be of concern if it led to differential recruitment according to factors related to arsenic exposure. Controls were selected at random from persons residing across the study area. Arsenic concentrations in well water are known to vary considerably within a small geographic area, with high variation for wells even less than 100 m apart (Van Geen et al. 2003). Concentrations also can vary considerably according to the depth of the well. In addition, we did not find an association between case status and ZIP code of residence (*p* = 0.3). Thus, we do not anticipate that potential geographic differences between cases and controls would explain our findings. Given the higher home ownership among controls, we also examined whether socioeconomic status could explain the variation seen in arsenic. Among controls, arsenic concentrations did not differ by home ownership (*p* = 0.2) or by income level (*p* = 0.9).

Other limitations of this study include self-reported demographic and medical information that is subject to the biases associated with that type of data collection. In addition, although participants had generally been living at the same address for a considerable time period (mean, 17 years), toenail arsenic concentrations represent exposures that occurred in the previous year, and our results should be considered in light of this limitation. Further, early life arsenic exposures may be potentially relevant to lung cancer development (Smith et al. 2006), and the retrospective design of this study prohibited us from collecting information on earlier exposures. We also did not have additional biomarkers, such as urinary arsenic or arsenic species (in urine or toenails), which could have shed additional light on individual arsenic methylation and subsequent cancer risk. An analysis of low-level arsenic exposure in Slovakia concluded that toenails were more predictive than urine of arsenic exposure at low concentrations (Wilhelm et al. 2005).

## Conclusions

We found associations between arsenic exposure and small-cell and squamous-cell carcinoma of the lung. These findings stress the relevance of evaluating the role of arsenic in lung cancer development in prospective studies of populations exposed to all arsenic levels. Further studies in persons at low levels of exposure, which include information on health history, would assist in modifying risk assessment for the U.S. population and elsewhere.

## Figures and Tables

**Table 1:** Characteristics of the study population and toenail arsenic concentrations.

Characteristic	Arsenic concentration (μg/g)						Crude odds of lung cancer associated with characteristic [OR (95% CI)]
	Cases (*n* = 223)			Controls (*n* = 238)			
	No. (%)	GM (SE)	*p*-Value	No. (%)	GM (SE)	*p*-Value	
Sex			0.3			0.8	

Male	100 (44.8)	0.063 (0.004)		97 (40.8)	0.073 (0.005)		1.00
Female	123 (55.2)	0.071 (0.006)		141 (59.2)	0.074 (0.004)		0.85 (0.58–1.22)

Age (years)			0.4			0.3	

30–49	24 (10.8)	0.081 (0.013)		34 (14.3)	0.076 (0.009)		1.00
50–59	56 (25.1)	0.071 (0.007)		60 (25.2)	0.079 (0.007)		1.32 (0.70– 2.50)
60–69	90 (40.4)	0.069 (0.006)		82 (34.5)	0.076 (0.006)		1.55 (0.85– 2.84)
≥ 70	53 (23.8)	0.054 (0.006)		62 (26.1)	0.063 (0.006)		1.21 (0.64– 2.29)

Race/ethnicity			0.3			0.4	

White non-Hispanic	213 (95.5)	0.087 (0.022)		233 (97.9)	0.058 (0.018)		1.00
All other ethnicities	10 (4.5)	0.066 (0.004)		5 (2.1)	0.076 (0.003)		2.19 (0.74– 6.50)

Educational attainment			0.09			0.1	

High school graduate or less	132 (59.2)	0.062 (0.004)		78 (32.8)	0.066 (0.005)		1.65 (1.37– 1.97)
Technical school, college, or more	91 (40.8)	0.074 (0.006)		160 (67.2)	0.077 (0.004)		1.00

BMI 6 months before interview			0.4			0.09	

17.2–24.9	89 (39.9)	0.062 (0.005)		77 (32.4)	0.081 (0.007)		1.00
25–29.9	72 (32.3)	0.073 (0.007)		78 (32.8)	0.071 (0.006)		0.80 (0.51–1.24)
≥ 30	62 (27.8)	0.066 (0.007)		83 (34.9)	0.068 (0.005)		0.65 (0.41–1.01)

Smoking (pack-years)			0.9			0.7	

0 (never-smoker)	12 (5.4)	0.065 (0.015)		100 (42.0)	0.072 (0.005)		1.00
< 20	19 (8.5)	0.064 (0.011)		69 (29.0)	0.080 (0.007)		2.29 (1.05–5.03)
21–40	49 (22.0)	0.072 (0.008)		36 (15.1)	0.064 (0.008)		11.3 (5.43–23.7)
≥ 41	143 (64.1)	0.065 (0.004)		33 (13.9)	0.075 (0.009)		36.1 (17.8–73.3)

Servings of fish (times per week)			0.03			0.03	

Never or < 1	89 (39.9)	0.057 (0.005)		88 (37.0)	0.070 (0.005)		1.00
1 to < 2	84 (37.7)	0.065 (0.005)		71 (29.8)	0.069 (0.006)		1.17 (0.76– 1.80)
2 to < 4	32 (14.4)	0.092 (0.013)		59 (24.8)	0.079 (0.007)		0.54 (0.32–0.90)
≥ 4	18 (8.1)	0.085 (0.015)		20 (8.4)	0.088 (0.014)		0.89 (0.44–1.79)

Selenium quartiles (toenail concentration, mg/kg)			0.6			0.01	

0.05–0.7644	96 (43.0)	0.066 (0.005)		59 (24.8)	0.056 (0.005)		1.00
0.7645–0.8901	48 (21.5)	0.062 (0.007)		60 (25.2)	0.077 (0.007)		0.49 (0.30– 0.81)
0.8902–1.075	47 (21.1)	0.066 (0.008)		60 (25.2)	0.076 (0.007)		0.48 (0.29– 0.79)
1.076–5.857	32 (14.3)	0.077 (0.011)		59 (24.8)	0.087 (0.008)		0.33 (0.19– 0.57)

Abbreviations: GM, geometric mean; CI, confidence interval. *p*-Values compare mean arsenic concentrations within groups.

**Table 2:** Toenail arsenic concentrations (μg/g) among study participants, by medical conditions and potential exposure routes.

Characteristic	Arsenic concentration (μg/g)						Crude odds of lung cancer associated with characteristic [OR (95% CI)]
	Cases (*n* = 223)			Controls (*n* = 238)			
	No. (%)	GM (SE)	*p*-Value	No. (%)	GM (SE)	*p*-Value	
Ever diagnosed with chronic bronchitis			0.2			0.3	

No	188	0.065 (0.004)		225	0.074 (0.003)		1.00
Yes	35	0.077 (0.010)		13	0.061 (0.012)		3.22 (1.66–6.27)

Ever diagnosed with COPD			0.6			0.2	

No	193	0.067 (0.004)		232	0.074 (0.003)		1.00
Yes	29	0.062 (0.009)		6	0.052 (0.015)		5.81 (2.36–14.3)

Ever diagnosed with fibrosis			0.5			0.2	

No	219	0.066 (0.004)		236	0.073 (0.003)		1.00
Yes	2	0.102 (0.057)		1	0.037 (0.026)		2.16 (0.19–23.9)

Ever diagnosed with asthma			0.3			0.4	

No	183	0.065 (0.004)		206	0.072 (0.004)		1.00
Yes	40	0.074 (0.009)		32	0.081 (0.010)		1.41 (0.85–2.33)

Ever diagnosed with any lung disease (bronchitis, COPD, or fibrosis)			0.8			0.09	

No	173	0.066 (0.004)		221	0.075 (0.004)		1.00
Yes	50	0.068 (0.008)		17	0.057 (0.010)		3.76 (2.09–6.74)

Ever diagnosed with diabetes			0.6			0.09	

No	192	0.067 (0.004)		216	0.075 (0.004)		1.00
Yes	31	0.062 (0.009)		22	0.059 (0.009)		1.59 (0.89–2.83)

History of cardiovascular disease (ever had coronary catheterization, angioplasty, heart attack, or stroke)			0.5			0.5	

No	183	0.067 (0.004)		200	0.074 (0.004)		1.00
Yes	40	0.062 (0.008)		38	0.068 (0.008)		1.15 (0.71–1.87)

Primary drinking water source			0.8			0.08	

Public water supply, bottled water drinker, or subject never drinks water	124	0.064 (0.005)		111	0.068 (0.005)		1.00
Well or spring water, filtered	34	0.063 (0.008)		50	0.070 (0.007)		0.61 (0.37–1.01)
Well or spring water, nonfiltered	64	0.074 (0.007)		77	0.084 (0.007)		0.75 (0.49–1.13)

Owns own home			0.03			0.2	

No	50	0.055 (0.006)		23	0.061 (0.009)		1.00
Yes	173	0.070 (0.004)		215	0.075 (0.004)		0.43 (0.27–0.68)

Ever employed in farmwork			0.5			0.02	

No	185	0.068 (0.004)		169	0.078 (0.004)		1.00
Yes	38	0.061 (0.008)		69	0.062 (0.005)		0.50 (0.32–0.79)

Ever employed in woodworking			0.1			0.5	

No	213	0.065 (0.003)		225	0.073 (0.003)		1.00
Yes	10	0.104 (0.026)		13	0.083 (0.016)		0.81 (0.35–1.89)

Abbreviations: GM, geometric mean; CI, confidence interval.

**Table 3:** Lung cancer in relation to lung disease and arsenic exposure.

	All lung cancers (*n* = 223)		Lung cancer cell types previously associated with arsenic (small cell and squamous cell) (*n* = 75)	
Measure	Cases/controls (*n*)	OR (95% CI)^a^	Cases/controls (*n*)	OR (95% CI)^a^
History of lung disease^b^				
Never	173/221	1.00	54/221	1.00
Ever	50/17	2.86 (1.39–5.91)	21/17	3.21 (1.25–8.24)
Toenail arsenic (μg/g)				
< 0.05	65/69	1.00	65/17	1.00
0.05 to < 0.0768	58/66	1.34 (0.71–2.53)	58/24	2.99 (1.12–7.99)
0.0768 to < 0.1137	58/44	1.10 (0.55–2.20)	58/13	1.86 (0.62–5.58)
≥ 0.1137	57/44	0.89 (0.46–1.75)	57/21	2.75 (1.00–7.57)

a Data are ORs and 95% confidence intervals (CIs) by logistic regression. Variables are adjusted for each other, as well as for sex, age, race/ethnicity, educational attainment, BMI, fish servings per week, smoking (pack-years), and selenium.

b Lung disease includes subjects who were ever diagnosed with bronchitis, COPD, or fibrosis.

**Table 4:** Toenail arsenic concentrations and lung cancer in relation to smoking and lung disease among cases (*n* = 223) and controls (*n* = 238).

Exposure	Arsenic concentration (μg/g)	All lung cancers	
		Cases/controls	OR (95% CI)
Smoking history^a^			
Never	< 0.05	4/32	1.00
Never	≥ 0.05	8/67	1.03 (0.28–3.75)
Ever	< 0.05	65/33	2.86 (0.83–9.80)
Ever	≥ 0.05	146/106	2.79 (0.87–8.94)
History of lung disease^b^			
Never	< 0.05	52/57	1.00
Never	≥ 0.05	121/164	1.02 (0.62–1.69)
Ever	< 0.05	17/8	1.31 (0.45–3.84)
Ever	≥ 0.05	33/9	4.78 (1.87–12.2)

a ORs and 95% confidence intervals (CIs) by logistic regression, with adjustment for sex, age, race/ethnicity, educational attainment, BMI, lung disease, fish servings per week, smoking (pack-years) and selenium.

b Model adjusts for sex, age, race/ethnicity, educational attainment, BMI, fish servings per week, smoking (pack-years) and selenium. Lung disease includes subjects who were ever diagnosed with bronchitis, COPD, or fibrosis.
